# Supplementary material for: Native Mass Spectrometry Analysis of Cullin RING Ubiquitin E3 Ligase Complexes in the Context of Targeted Protein Degradation
Source: bioRxiv. 2026 Jan 7:2025.12.04.692288. Preprint. [Version 2] doi: 10.64898/2025.12.04.692288 (PMC12803233; doi:10.64898/2025.12.04.692288)
Supplement: Supplement 1 [file media-1.pdf]

SUPPORTING INFORMATION

Native Mass Spectrometry Analysis of Cullin RING Ubiquitin E3 Ligase Complexes in the Context of Targeted Protein Degradation

Louise M Sternicki[1,*], Charlotte Crowe[2], Lianne H. E. Wieske[2], Alessio Ciulli[2], Sally-Ann Poulsen[1,*]

*Email: l.sternicki@griffith.edu.au; s.poulsen@griffith.edu.au

Affiliations

[1]Institute for Biomedicine and Glycomics, Griffith University, Gold Coast, Queensland 4222, Australia

[2]Centre for Targeted Protein Degradation, School of Life Sciences, University of Dundee, 1 James Lindsay Place, Dundee DD1 5JJ, United Kingdom

Table of Contents

Supporting Table S1. Sequences and calculated molecular weight (MW) of protein constructs utilised in this study. S4

Supporting Figure S1. Raw and deconvoluted nMS spectra for individual protein components. S7

Supporting Table S2. Observed *m/z* values, charge states and measured MWs for individual protein components. S8

Supporting Table S3. Reference corrected MW of protein components, PROTAC/compound MWs and expected MW of protein complexes determined (based on corrected MW). S9

Supporting Figure S2. Collision-induced dissociation (CID) of minimal E3 (VCB) and full E3 ($CRL2^{VHL}$) into individual component subunits. S10

Supporting Table S4. Observed MW and assignment to subunits dissociated from E3s VCB and $CRL2^{VHL}$ with in-source CID. S11

Supporting Table S5. Observed *m/z* values and measured MWs for binary binding of MZ1 (MW 1002.6 Da) and *cis*-MZ1 (MW 1002.6 Da) to $BRD4^{BD2}$, VCB and $CRL2^{VHL}$. S12

Supporting Table S6. Observed *m/z* values and measured MWs for samples comprising $BRD4^{BD2}$ and either VCB or $CRL2^{VHL}$ with MZ1 (MW 1002.6 Da) or *cis*-MZ1 (MW 1002.6 Da). S14

Supporting Figure S3. nMS titration of PROTAC MZ1 (1.0-2.0 equiv, 0.1 increments) against POI $BRD4^{BD2}$ and E3 VCB (present at equimolar concentrations, 1 μM each) to determine the optimal MZ1 equiv for maximal ternary complex formation. S17

Supporting Figure S4. Quantification of ternary complex formation between $BRD4^{BD2}$ and VCB (present at equimolar concentrations, 1 μM) with MZ1 titrated (1.0-2.0 equiv, 0.1 increments) as measured by nMS. S18

Supporting Figure S5. Raw data nMS spectra of E3-E2-Ub sample comprising $CRL2^{VHL}$ (1 μM) and UBE2R1(C93K)-Ub (5 μM). S19

Supporting Table S7. Observed *m/z* values, charge states and measured MWs for interacting species in the nMS spectra of the E3-E2-Ub sample ($CRL2^{VHL}$ and UBE2R1(C93K)-Ub). S20

Supporting Figure S6. Raw data nMS spectra showing PROTAC mediated formation of the full $BRD4^{BD2}$-MZ1-$CRL2^{VHL}$-UBE2R1(C93K)-Ub complex. S21

Supporting Table S8. Observed *m/z* values and measured MWs for the full POI-PROTAC-E3-E2-Ub degradation complex. S22

Supporting Figure S7. nMS assessment of KRAS and binary binding with ACBI3 and *cis*-ACBI3. ....S24

Supporting Table S9. Observed *m/z* values and measured MWs for binary binding of ACBI3 (MW 1019.3 Da) and *cis*-ACBI3 (MW 1019.3 Da) to KRAS, VCB and $CRL2^{VHL}$. S25

Supporting Table S10. Expected MW of KRAS protein complexes (based on corrected MW). S27

Supporting Figure S8. nMS analysis of ternary complex POI-PROTAC-E3 formation between KRAS (1 μM, 1 equiv), ACBI3 (1.9 μM, 1.9 equiv) and A) VCB (1 μM, 1 equiv) or B (1 μM, 1 equiv). S28

Supporting Table S11. Observed *m/z* values and measured MWs for samples comprising KRAS and either VCB or $CRL2^{VHL}$ with ACBI3 (MW 1019.3 Da) and *cis*-ACBI3 (MW 1019.3 Da). S29

Supporting Figure S9. nMS titration of PROTAC ACBI3 (0.8-2.5 equiv, 0.1 increments) against POI KRAS and E3 VCB (present at equimolar concentrations, 1 μM each) to determine the optimal ACBI3 equiv for maximal ternary complex formation. S31

Supporting Figure S10. Quantification of ternary complex formation between KRAS and VCB (present at equimolar concentrations, 1 μM each) with ACBI3 titrated (0.8-2.5 equiv, 0.1 increments) as measured by nMS. S32

Supporting Figure S11. Raw data nMS spectra showing PROTAC mediated formation of the full KRAS-ACBI3-CRL2^{VHL}-UBE2R1(C93K)-Ub complex. S33

Supporting Table S12. Observed *m/z* values and measured MWs for the full POI-PROTAC-E3-E2-Ub degradation complex with KRAS as the POI. S34

Recombinant production of CRL2^{VHL} protein. S36

Recombinant production of KRAS protein S37

Supporting Table S13. UHMR MS parameters employed for the methods used to characterise proteins and/or complexes. S39

References S41

Table S1. Sequences and calculated molecular weight (MW) of protein constructs utilised in this study.

Protein	Subunit/s	Sequence	Subunit/s MW	Protein/protein complex MW
BRD4^{BD2} (residues 333-460)	N/A	SMKDVPDSQQHPAPEKSSKVSEQLKCCSGILKEMF AKKHAAYAWPFYKPVDVEALGLHDYCDIIKHPMDM STIKSKLEAREYRDAQEFGADVRLMFSNCYKYNPP DHEVVAMARKLQDVFEMRFAKMPDE	N/A	15,036.3
KRAS (residues 1-169, GDP-bound form)	N/A	HMTEYKLVVVGAGGVGKSALTIQLIQNHFVDEYDP TIEDSYRKQVVIDGETCLLDILDTAGQEEYSAMRDQ YMRTGEGFLCVFAINNTKSFEDIHHYREQIKRVKDSE DVPMVLVGNKCDLPSRTVDTKQAQDLARSYGIPFIE TSAKTRQGVDDAFYTLVREIRKHKEK	N/A	19,850.4[a]
VCB (VHL-EloC-EloB)	VHL (residues 54-213)	GSMEAGRPRPVLRSVNSREPSQVIFCNRSPRVVL PVWLNFDGEPQPYPTLPPGTGRRIHSYRGHLWLF RDAGTHDGLLVNQTELFVPSLNVDGQPIFANITLP VYTLKERCLQVVRSLVKPENYRRLDIVRSLYEDLE DHPNVQKDLERLTQERIAHQRMGD	18,676.3	41,373.3
	EloC (residues 1-104)	MDVFLMIRRHKTTIFTDAKESSTVFELKRIVEGILK RPPDEQRLYKDDQLLDDGKTLGECGFTSQTARP QAPATVGLAFRADDTFEALCIEPFSSPPELPDVMK	11,733.4	
	EloB (residues 17-112)	MMYVKLISSDGHEFIVKREHALTSGTIKAMLSGP GQFAENETNEVNFREIPSHVLSKVCMYFTYKVR YTNSSTEIPEFPIAPEIALELLMAANFLDC	10,963.6	
CRL2^{VHL} (VHL-EloC-EloB-Cul2-Rbx1)	VHL	GSMEAGRPRPVLRSVNSREPSQVIFCNRSPRVVL PVWLNFDGEPQPYPTLPPGTGRRIHSYRGHLWLF	18,676.3	140,831.5

(residues 54-213)	RDAGTHDGLLVNQTELFVPSLNVDGQPIFANITLP VYTLKERCLQVVRSLVKPENYRRLDIVRSLYEDLE DHPNVQKDLERLTQERIAHQRMGD	
EloC (residues 1-104)	MDVFLMIRRHKTTIFTDAKESSTVFELKRIVEGILK RPPDEQRLYKDDQLLDDGKTLGECGFTSQTARP QAPATVGLAFRADDTFEALCIEPFSSPPELPDVMK	11,733.4
EloB (residues 17-112)	MMYVKLISSDGHEFIVKREHALTSGTIKAMLSGP GQFAENETNEVNFREIPSHVLSKVCMYFTYKVR YTNSSTEIPEFPIAPEIALELLMAANFLDC	10,963.6
Cul2 (residues 1-745)	GGSMSLKPRVVDFDETWNKLLTTIKAVVMLEYVER ATWNDRFSDIYALCVAYPEPLGERLYTETKIFLENH VRHLHKRVLESEEQVLVMYHRYWEEYSKGADYMD CLYRYLNTQFIKKNKLTEADLQYGYGGVDMNEPLM EIGELALDMWRKLMVEPLQAILIRMLLREIKNDRGG EDPNQKVIHGVINSFVHVEQYKKKFPLKFYQEIFES PFLTETGEYYKQEASNLLQESNCSQYMEKVLGRLK DEEIRCRKYLHPSSYTKVIHECQQRMVADHLQFLH AECHNIIRQEKKNDMANMYVLLRAVSTGLPHMIQEL QNHIHDEGLRATSNLTQENMPTLFVESVLEVHGKF VQLINTVLNGDQHFMSALDKALTSVVNYREPKSVC KAPELLAKYCDNLLKKSAKGMTENEVEDRLTSFITV FKYIDDKDVFQKFYARMLAKRLIHGLSMSMDSEEA MINKLKQACGYEFTSKLHRMYTDMSVSADLNNKF NNFIKNQDTVIDLGISFQIYVLQAGAWPLTQAPSST FAIPQELEKSVQMFELFYSQHFSGRKLTWLHYLCT GEVKMNYLGKPYVAMVTTYQMAVLLAFNNSETVS YKELQDSTQMNEKELTKTIKSLLDVKMINHDSEKE DIDAESSFSLNMNFSSKRTKFKITTSMQKDTPQEM	87,184.2

		EQTRSAVDEDRKMYLQAAIVRIMKARKVLRHNALI<br>QEVISQSRARFNPSISMIKKCIEVLIDKQYIERSQAS<br>ADEYSYVA		
	Rbx1<br>(residues 1-108)	MAAAMDVDTPSGTNSGAGKKRFEVKKWNAVALW<br>AWDIVVDNCAICRNHIMDLCIECQANQASATSEEC<br>TVAWGVCNHAFHFHCISRWLKTRQVCPLDNREW<br>EFQKYGH	12,274.0	
UBE2R1<br>(C93K)	N/A	ARPLVPSSQKALLLELKGLQEEPVEGFRVTLVDE<br>GDLYNWEVAIFGPPNTYYEGGYFKARLKFPIDYP<br>YSPPAFRFLTKMWHPNIYETGDVKISILHPPVDDP<br>QSGELPSERWNPTQNVRTILLSVISLLNEPNTFS<br>PANVDASVMYRKWKESKGKDREYTDIIRKQVLG<br>TKVDAERDGVKVPTTLAEYCVKTKAPAPDEGSD<br>LFYDDYYEDGEVEEEADSCFGDDEDDSGTEES	N/A	26,630.6
UBE2RI<br>(C93K)-^{15}N-Ub	UBE2R1<br>(C93K)	ARPLVPSSQKALLLELKGLQEEPVEGFRVTLVDE<br>GDLYNWEVAIFGPPNTYYEGGYFKARLKFPIDYP<br>YSPPAFRFLTKMWHPNIYETGDVKISILHPPVDDP<br>QSGELPSERWNPTQNVRTILLSVISLLNEPNTFS<br>PANVDASVMYRKWKESKGKDREYTDIIRKQVLG<br>TKVDAERDGVKVPTTLAEYCVKTKAPAPDEGSD<br>LFYDDYYEDGEVEEEADSCFGDDEDDSGTEES	26,630.6	35,283.4[b]
	^{15}N-Ub	MQIFVKTLTGKTITLEVEPSDTIENVKAKIQDKEG<br>IPPDQQRLIFAGKQLEDGRTLSDYNIQKESTLHL<br>VLRLRGG	8,670.8	

[a]Expected MW calculated considers the protein sequence (19,382.9 Da) and bound Guanosine 5′-diphosphate (GDP, 443.2 Da) and Mg^{2+} (24.3 Da). [b]Expected MW calculated considers the covalent bond between E2 and Ub (Δ -18 Da).

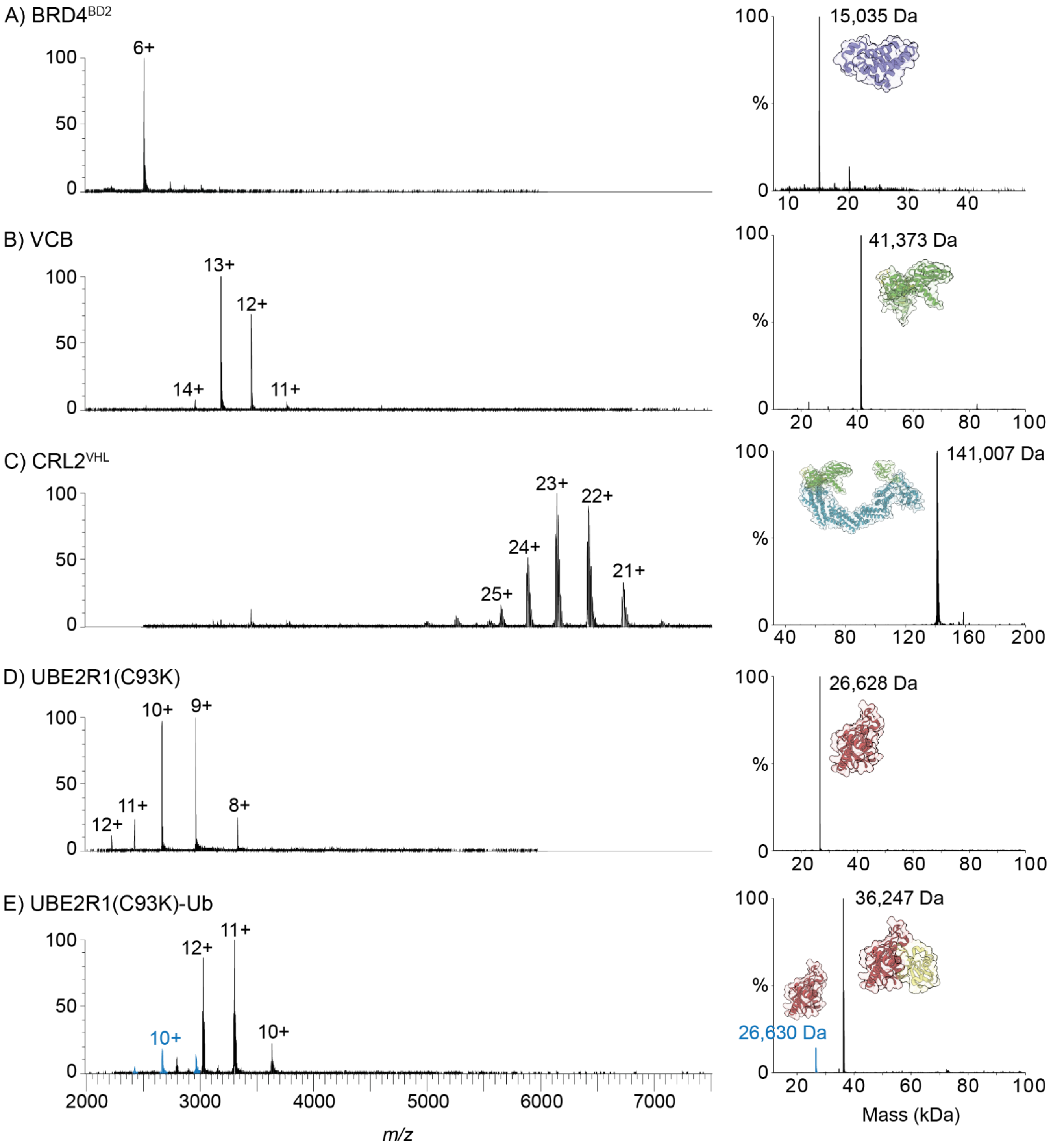

Supporting Figure S1. Raw and deconvoluted nMS spectra for individual protein components (150 mM NH_4OAc). A) 1 µM BRD4^{BD2}, expected MW is 15,036 Da. B) 1 µM VCB, expected MW is 41,373 Da. C) 1.5 µM CRL2^{VHL}, expected MW is 140,832 Da (or ~141,023 Da with 3× Zn^{2+} cations as observed in PDB 8RWX and PDB 8RX0). D) 1 µM UBE2R1(C93K), expected MW is 26,631 Da. E) 1 µM UBE2R1(C93K)-Ub, expected MW is 35,283Da (^{15}N-labeled Ub is used, referred to as 'Ub' throughout remaining analysis). The blue peaks and labels in E) correspond to a minor (~9.6%) amount of E2 with no Ub conjugated. Observed *m/z* values and MWs in **Supporting Tables S2** and **S3**.

Supporting Table S2. Observed *m/z* values, charge states and measured MWs for individual protein components. Raw and deconvoluted mass spectra are shown in **Supporting Figure S1**.

**Protein Component**	**Expected MW (Da)**	**Observed *m/z***	**Charge state (*z*)**	**Deconvoluted MW (Da)**[a]
BRD4^{BD2}	15,036.3	2506.8765	6+	15,035
VCB (VHL-EloC-EloB)	41,373.4	2956.3498	14+	41,373
		3183.5237	13+	
		3448.6302	12+	
		3762.0383	11+	
CRL2^{VHL} (VHL-EloC-EloB-Cul2-Rbx1)	140,831.5 (or ~141,023 Da with 3× Zn^{2+} cations as observed in PDB 8RWX and PDB 8RX0)	5641.4632	25+	141,007
		5876.6072	24+	
		6132.4421	23+	
		6411.3233	22+	
		6716.9335	21+	
		7052.9235	20+	
UBE2R1(C93K)	26,630.6	2219.9264	12+	26,628
		2421.7416	11+	
		2663.818	10+	
		2959.6957	9+	
		3329.5262	8+	
UBE2R1(C93K)-^{15}N-Ub	35,283.5[b]	2421.8754	11+	26,630[c]
		2663.9924	10+	
		2959.8884	9+	
		2789.2336	13+	36,247
		3021.6625	12+	
		3296.2424	11+	
		3625.7357	10+	

[a]MW deconvoluted in UniDec,[2] to nearest whole Da. [b]Expected MW calculated considers the covalent bond between E2 and Ub (Δ -18 Da). [c]Corresponds to a minor (~9.6%) amount of E2 with no Ub conjugated.

Supporting Table S3. Reference corrected MW of protein components, PROTAC/compound MWs and expected MW of protein complexes determined (based on corrected MW).

**Protein**	**Corrected MW (Da)**[a]
BRD4^{BD2}	15,036[b]
VCB	41,373[b]
CRL2^{VHL}	141,007[c]
UBE2R1(C93K)	26,631[b]
UBE2R1(C93K)-Ub	36,247[c]
**Compounds/PROTACs**	**MW (Da)**
MZ1	1002.6
cis-MZ1	1002.6
**Protein Complexes**	**Expected MW (Da)**[d]
BRD4^{BD2}-VCB[e]	56,409
BRD4^{BD2}-MZ1-VCB[f]	57,412
BRD4^{BD2}-CRL2^{VHL}[e]	156,043
BRD4^{BD2}-MZ1-CRL2^{VHL}[f]	157,046
CRL2^{VHL}-UBE2R1(C93K)	167,638
CRL2^{VHL}-UBE2R1(C93K)-Ub	177,254
BRD4^{BD2}-CRL2^{VHL}-UBE2R1(C93K)-Ub[e]	192,290
BRD4^{BD2}-MZ1-CRL2^{VHL}-UBE2R1(C93K)-Ub[f]	193,293

[a]Corrected MW is based on the nMS analysis for protein components (**Supporting Figure S1**) and are used for data analysis throughout the rest of the study. [b]MW as determined from protein sequence (corresponds with MW as measured by nMS, **Supporting Figure S1**). [c]MW as determined from nMS visibility studies (**Supporting Figure S1**) due to differences with that expected from protein sequence. [d]Expected MWs of protein complexes of interest for this study, calculated using the corrected MWs for individual protein components (rounded to nearest Da). [e]Complexes without PROTAC and not necessarily expected to form, but MWs provided to compare with spectra to confirm presence or absence. [f]Complexes containing *cis*-MZ1 are not expected to form, however, due to the MW of *cis*-MZ1, any such complexes would have the same MW as the MZ1-containing complexes.

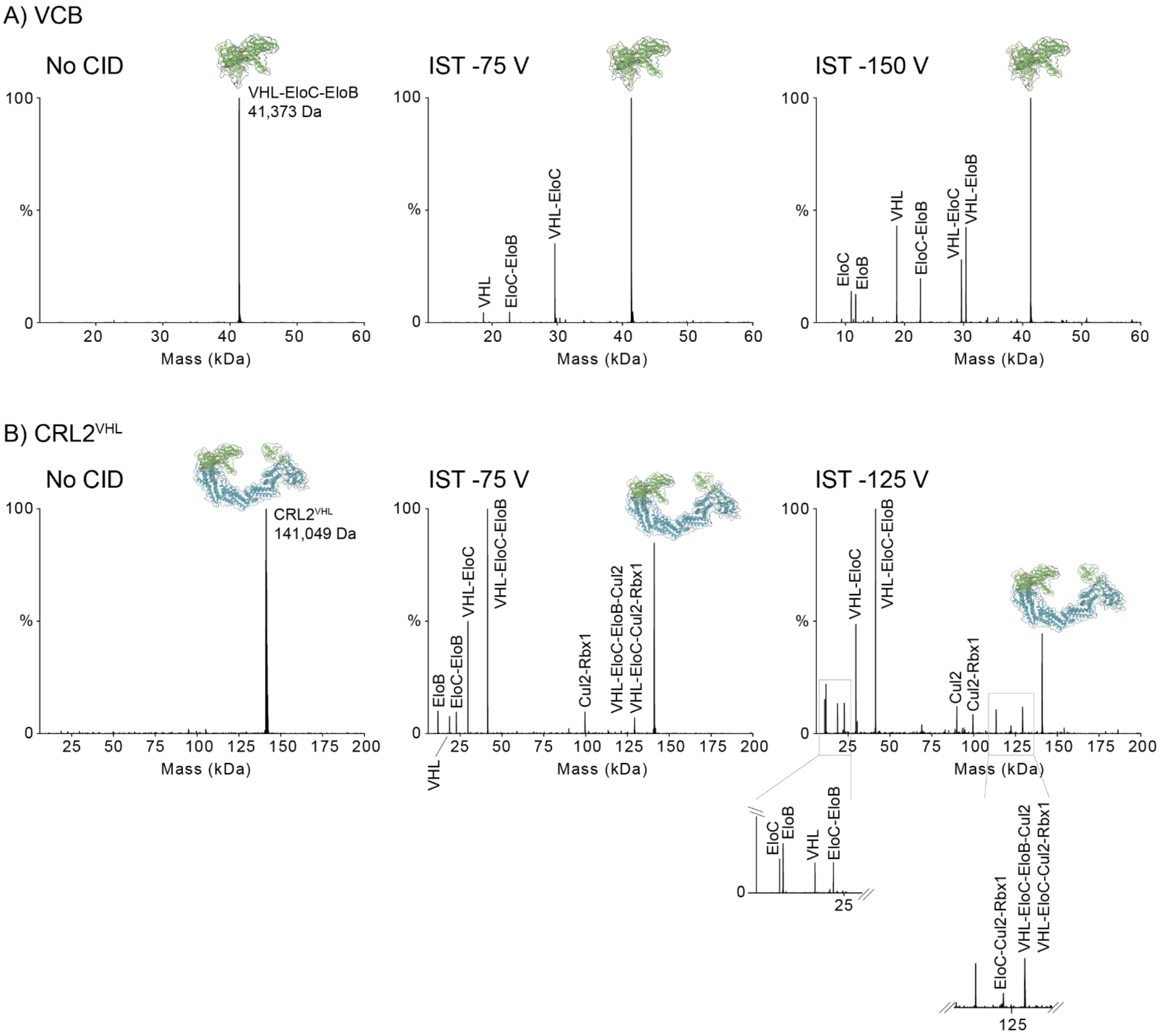

Supporting Figure S2. Collision-induced dissociation (CID) of minimal E3 (VCB) and full E3 ($CRL2^{VHL}$) into individual component subunits. A) VCB (1 µM) analysis without CID and with subunit dissociation via in-source trapping desolvation voltages -75 V and -150 V. B) $CRL2^{VHL}$ (1 µM) analysis without CID and with subunit dissociation via in-source trapping desolvation voltages -75 V and -125 V ($CRL2^{VHL}$ was not visible at -150 V for a direct comparison with VCB analysis). Deconvoluted spectra are annotated with the dissociated component identities; unlabelled peaks were unable to be assigned to a known intact subunit/s. Observed MWs are provided in **Supporting Table S4.**

Supporting Table S4. Observed MW and assignment to subunits dissociated from E3s VCB and CRL2^{VHL} with in-source CID. Deconvoluted mass spectra are in **Supporting Figure S2**.

Protein	CID V	Figure/Spectra	Observed MW (Da)[a]	Assigned Subunits	Expected MW (Da)
VCB	0	S2A, left	41,373	VHL-EloC-EloB	41,373
	-75	S2A, middle	18,675	VHL	18,676
			22,696	EloC-EloB	22,697
			29,639	VHL-EloC	29,640
			41,373	VHL-EloC-EloB	41,373
	-150	S2A, right	10,962	EloC	10,964
			11,732	EloB	11,733
			18,675	VHL	18,676
			22,695	EloC-EloB	22,697
			29,639	VHL-EloC	29,640
			30,408	VHL-EloB	30,410
			41,372	VHL-EloC-EloB	41,373
CRL2^{VHL}	0	S2B, left	141,049	VHL-EloC-EloB-Cul2-Rbx1	140,832
	-75	S2B, middle	11,732	EloB	11,733
			18,676	VHL	18,676
			22,697	EloC-EloB	22,697
			29,638	VHL-EloC	29,640
			41,369	VHL-EloC-EloB	41,373
			99,583	Cul2-Rbx1	99,458
			129,257	VHL-EloC-EloB-Cul2 or VHL-EloC-Cul2-Rbx1	128,558 or 129,098
			141,000	VHL-EloC-EloB-Cul2-Rbx1	140,832
	-125	S2B, right	10,962	EloC	10,964
			11,731	EloB	11,733
			18,676	VHL	18,676
			22,696	EloC-EloB	22,697
			29,637	VHL-EloC	29,640
			30,408	VHL-EloB	30,410
			41,370	VHL-EloC-EloB	41,373
			68,996	[b, c]	
			89,954	Cul2	87,184
			99,575	Cul2-Rbx1	99,458
			113,455	[b]	
			122,365	EloC-EloB-Cul2-Rbx1[c]	122,155
			129,239	VHL-EloC-EloB-Cul2 or VHL-EloC-Cul2-Rbx1	128,558 or 129,098
			140,971	VHL-EloC-EloB-Cul2-Rbx1	140,832

[a]MW deconvoluted in UniDec,[2] to nearest whole Da. [b]Observed MW unable to be assigned to a known intact subunit/s. [c]Protein complexes were observed at relative intensity <5%.

Supporting Table S5. Observed *m/z* values and measured MWs for binary binding of MZ1 (MW 1002.6 Da) and *cis*-MZ1 (MW 1002.6 Da) to $BRD4^{BD2}$, VCB and $CRL2^{VHL}$. Raw mass spectra (of the most intense charge states) are shown in **Figure 3**. Binary complexes – blue text.

Sample: Protein	Sample: Ligand	Protein Species	Expected MW (Da)	Observed *m/z*	Charge State (*z*)	Deconvoluted MW (Da)[a]	Bound Ligand MW (ΔMW) (Da)
$BRD4^{BD2}$	-	$BRD4^{BD2}$	15,036.3	2506.8596<br>3007.8315	6+<br>5+	15,035	N/A
	MZ1	$BRD4^{BD2}$	15,036.3	2506.6916<br>3008.0313	6+<br>5+	15,034	N/A
		$BRD4^{BD2}$-MZ1	16,038.9	2292.2146<br>2674.2514	7+<br>6+	16,038	1,002
	cis-MZ1	$BRD4^{BD2}$	15,036.3	2507.0247<br>3008.4322	6+<br>5+	15,036	N/A
		$BRD4^{BD2}$-*cis*-MZ1	16,038.9	2291.9269<br>2674.0825	7+<br>6+	16,038	1,002
VCB	-	VCB	41,373.4	3448.5599<br>3762.1009<br>4138.1978<br>4597.819	12+<br>11+<br>10+<br>9+	41,373	N/A
	MZ1	VCB	41,373.4	3448.5423<br>3762.0871<br>4138.1848<br>4597.8366	12+<br>11+<br>10+<br>9+	41,373	N/A
		VCB-MZ1	42,376.0	3532.2241<br>3853.1851<br>4238.3755<br>4709.4314	12+<br>11+<br>10+<br>9+	42,375	1,002
	cis-MZ1	VCB	41,373.4	3448.622<br>3762.1614<br>4138.2476<br>4597.9403	12+<br>11+<br>10+<br>9+	41,373	N/A
$CRL2^{VHL}$	-	$CRL2^{VHL}$	141,007	6412.0768<br>6717.8703<br>7053.887<br>7425.1089<br>7837.7589	22+<br>21+<br>20+<br>19+<br>18+	141,060	N/A
	MZ1	$CRL2^{VHL}$	141,007	6411.0288<br>6719.9109<br>7053.098<br>7424.2263	22+<br>21+<br>20+<br>19+	141,039	N/A

			7837.1655	18+		
	CRL2^{VHL}-MZ1	142,010	6456.9744	22+	142,035	996
			6764.1866	21+		
			7103.0769	20+		
			7476.8274	19+		
			7892.6892	18+		
cis-MZ1	CRL2^{VHL}	141,007	6411.3432	22+	141,042	N/A
			6717.0904	21+		
			7053.5664	20+		
			7424.9112	19+		
			7837.5369	18+		

[a]MW deconvoluted in UniDec,[2] to nearest whole Da and with relative intensity >5-10%. N/A: not applicable as no ligand binding observed.

Supporting Table S6. Observed *m/z* values and measured MWs for samples comprising BRD4^{BD2} and either VCB or CRL2^{VHL} with MZ1 (MW 1002.6 Da) and *cis*-MZ1 (MW 1002.6 Da). Raw mass spectra as shown in **Figure 4**. Binary complexes – blue text, ternary complexes – red text.

Sample POI	E3	Compound	Observed Species	Expected MW (Da)	Observed *m/z*	Charge State (*z*)	Deconvoluted MW (Da)[a]
BRD4^{BD2}	VCB	-	BRD4^{BD2}	15,036.3	2506.7104	6+	15,034
			VCB	41,373.4	3448.6663	12+	41,372
					3762.116	11+	
					4138.1691	10+	
					4597.8007	9+	
		MZ1	BRD4^{BD2}	15,036.3	2506.6251	6+	[b]
			BRD4^{BD2}-MZ1	16,038.9	2292.1765	7+	[b]
					2673.8921	6+	
			VCB	41,373.4	3448.5386	12+	41,372
					3762.0472	11+	
					4138.1602	10+	
			VCB-MZ1	42,376.0	3532.1979	12+	42,374
					3853.1694	11+	
					4238.3828	10+	
			BRD4^{BD2}-MZ1-VCB	57,412.3	3828.3368	15+	57,409
					4101.6456	14+	
					4417.0471	13+	
					4784.988	12+	
					5220.0705	11+	
		cis-MZ1	BRD4^{BD2}	15,036.3	2506.6498	6+	15,037
			BRD4^{BD2}-*cis*-MZ1	16,038.9	2291.9968	7+	16,038
					2673.8488	6+	
			VCB	41,373.4	3448.6715	12+	41,372
					3762.1097	11+	
					4138.1529	10+	
					4597.7988	9+	
	CRL2^{VHL}	-	BRD4^{BD2}	15,036.3	2149.0475	7+	15,036
					2506.8536	6+	
					3008.0689	5+	
			VCB	41,373.4	3448.6337	12+	41,371
					3761.9333	11+	
					4138.4009	10+	
					4598.1230	9+	
			Cul2-Rbx1	99,458.2	5533.3442	18+	99,590
					5859.2324	17+	
					6225.5383	16+	
					6640.5519	15+	
			CRL2^{VHL}	141,007	6409.2994	22+	140,996

			6714.8612	21+	
			7050.951	20+	
			7422.4237	19+	
			7835.0047	18+	
			8297.3235	17+	
MZ1	$BRD4^{BD2}$	15,036.3	2148.877	7+	15,036
			2506.8293	6+	
	VCB	41,373.4	3448.6723	12+	41,369
			3761.8423	11+	
			4137.836	10+	
			4597.8113	9+	
	$BRD4^{BD2}$-VCB	56,409.7	4340.2257	13+	56,411
			4701.9928	12+	
			5129.4489	11+	
	$BRD4^{BD2}$-MZ1-VCB	57,412.3	4417.4739	13+	57,415
			4785.5243	12+	
			5220.6368	11+	
	Cul2-Rbx1	99,458.2	5533.2309	18+	99,580
			5858.6474	17+	
			6224.9399	16+	
			6639.9786	15+	
	$CRL2^{VHL}$	141,007.0	6409.344	22+	140,999
			6714.964	21+	
			7051.527	20+	
			7422.7996	19+	
			7835.0374	18+	
	$BRD4^{BD2}$-MZ1-$CRL2^{VHL}$	157,045.6	6543.2716	24+	157,069
			6828.7287	23+	
			7140.0304	22+	
			7480.2482	21+	
			7854.3152	20+	
			8268.4585	19+	
			8728.082	18+	
cis-MZ1	$BRD4^{BD2}$	15,036.3	2157.5124	7+	15,035
			2506.8337	6+	
			3008.1005	5+	
	VCB	41,373.4	3448.6355	12+	41,373
			3762.1336	11+	
			4138.336	10+	
	Cul2-Rbx1	99,458.2	5534.6643	18+	99,611
			5860.1552	17+	
			6227.1531	16+	
	$CRL2^{VHL}$	141,007	6411.263	22+	141,038
			6716.9436	21+	
			7052.8503	20+	
			7424.2839	19+	

		7837.3589	18+	
		8299.3488	17+	

[a]MW deconvoluted in UniDec,[2] to nearest whole Da and with relative intensity >5%. [b]Species not detected during deconvolution but evident in raw spectrum.

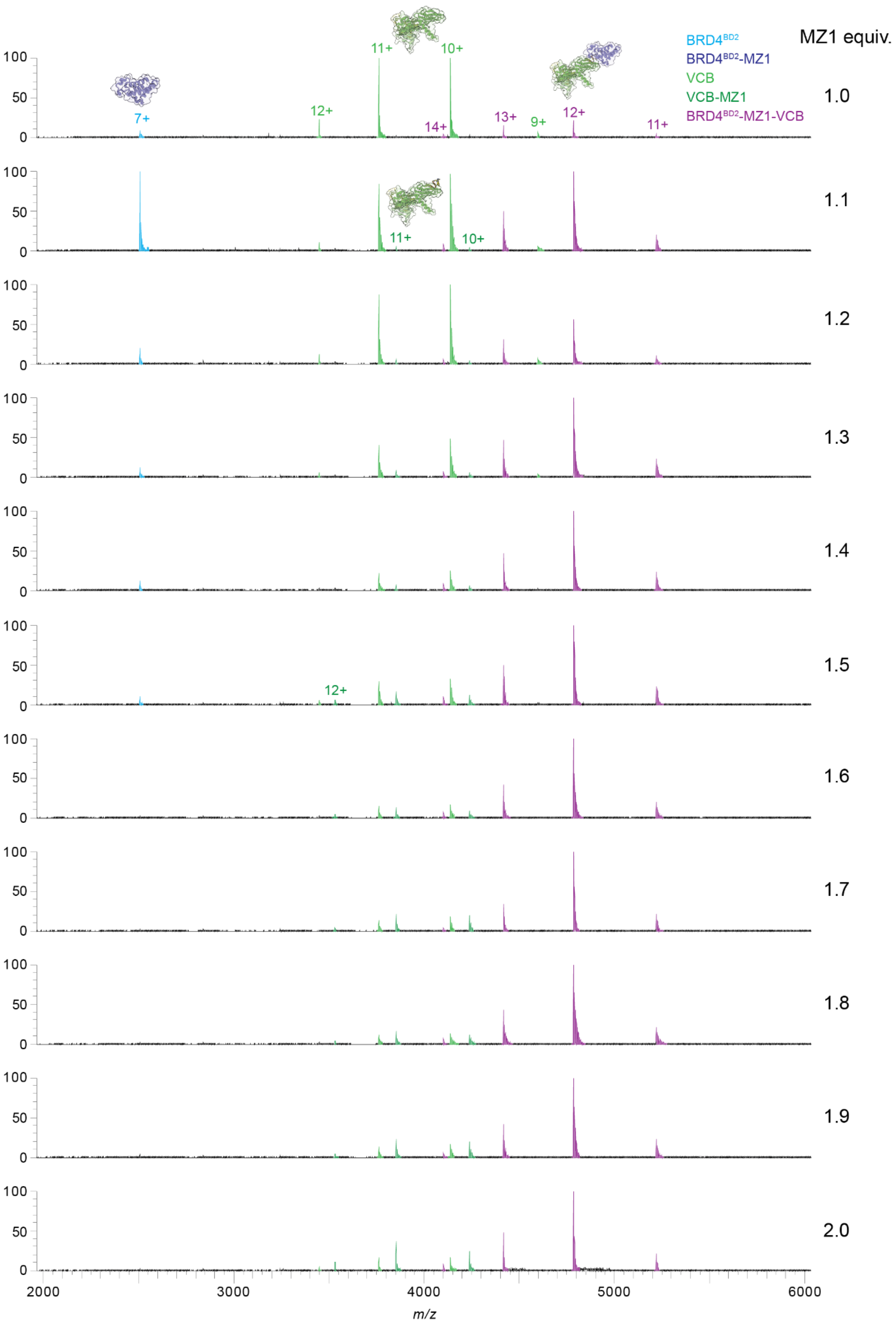

Supporting Figure S3. nMS titration of PROTAC MZ1 (1.0-2.0 equiv, 0.1 increments) against POI BRD4^{BD2} and E3 VCB (present at equimolar concentrations, 1 μM each) to determine the optimal MZ1 equiv for maximal ternary complex formation.

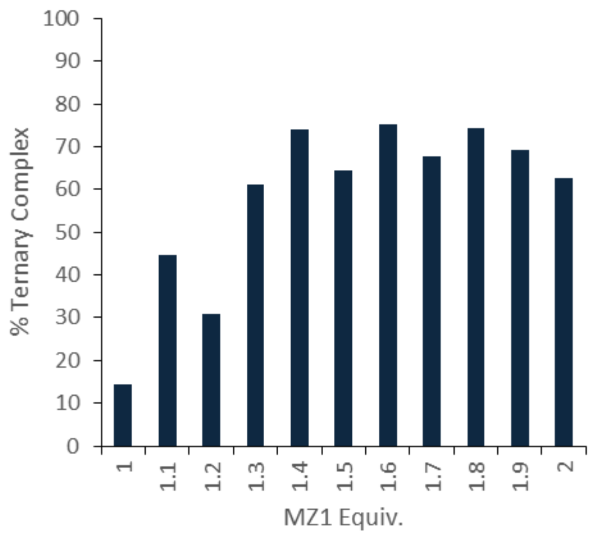

Supporting Figure S4. Quantification of ternary complex formation between $BRD4^{BD2}$ and VCB (present at equimolar concentrations, 1 µM each) with MZ1 titrated (1.0-2.0 equiv, 0.1 increments) as measured by nMS. The % ternary complex formed was calculated from the deconvoluted data of mass spectra in **Supporting Figure S3** as the ratio of VCB recruited into the ternary complex (i.e. sum of the intensity of the deconvoluted ternary complex peak divided by the sum of the intensities of the deconvoluted peaks for unbound VCB, MZ1-bound VCB and ternary complex).

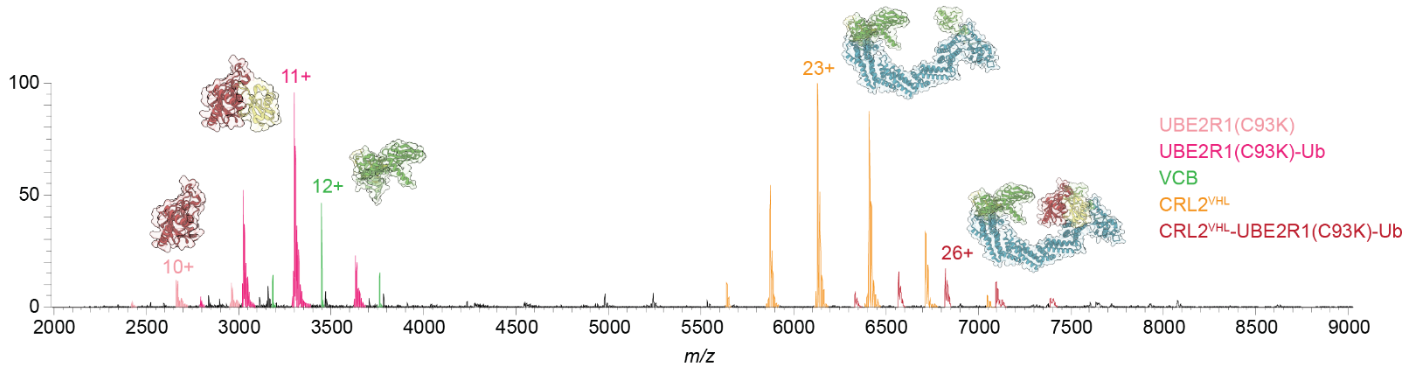

Supporting Figure S5. Raw data nMS spectra of E3-E2-Ub sample comprising CRL2^{VHL} (1 µM) and UBE2R1(C93K)-Ub (5 µM). E3-E2-Ub complex (CRL2^{VHL}-UBE2R1(C93K)-Ub, red) was detected, along with free CRL2^{VHL} (orange), dissociated VCB (green), free UBE2R1(C93K)-Ub (dark pink, and contaminating UBE2R1(C93K) without Ub conjugated, light pink). Where the deconvoluted species was present at >5% relative intensity the peaks are labelled, including with the charge state for the most intense peak for each observed species. Corresponding deconvoluted nMS are in manuscript **Figure 5A** and observed *m/z* values and MWs in **Supporting Table S7**

Supporting Table S7. Observed *m/z* values, charge states and measured MWs for species observed in the nMS spectra of the E3-E2-Ub sample (CRL2^{VHL} and UBE2R1(C93K)-Ub). Raw and deconvoluted mass spectra are shown in **Supporting Figure S5** and **Figure 5A**, respectively.

**Sample**	**Protein Species**	**Expected MW (Da)**	**Observed *m/z***	**Charge State (z)**	**Deconvoluted MW (Da)^a**
CRL2^{VHL}, UBE2R1(C93K)-Ub	UBE2R1(C93K)	26,630.6	2421.7311	11+	26,630
			2664.0343	10+	
			2959.9475	9+	
	UBE2R1(C93K)-Ub	36,247.0	2789.265	13+	36,308
			3021.7972	12+	
			3296.3306	11+	
			3625.7973	10+	
	VHL-EloC-EloB	41,373.4	3183.5315	13+	41,370
			3448.4197	12+	
			3762.0578	11+	
	CRL2^{VHL}	141,007.0	5639.5119	25+	140,969
			5874.7173	24+	
			6130.2455	23+	
			6409.0395	22+	
			6713.9964	21+	
			7049.544	20+	
	CRL2^{VHL}-UBE2R1(C93K)-Ub	177,254.0	6333.9635	28+	177,339
			6568.7772	27+	
			6821.5043	26+	
			7095.0852	25+	
			7390.1709	24+	

^{a}MW deconvoluted in UniDec,[2] to nearest whole Da and with relative intensity >5%.

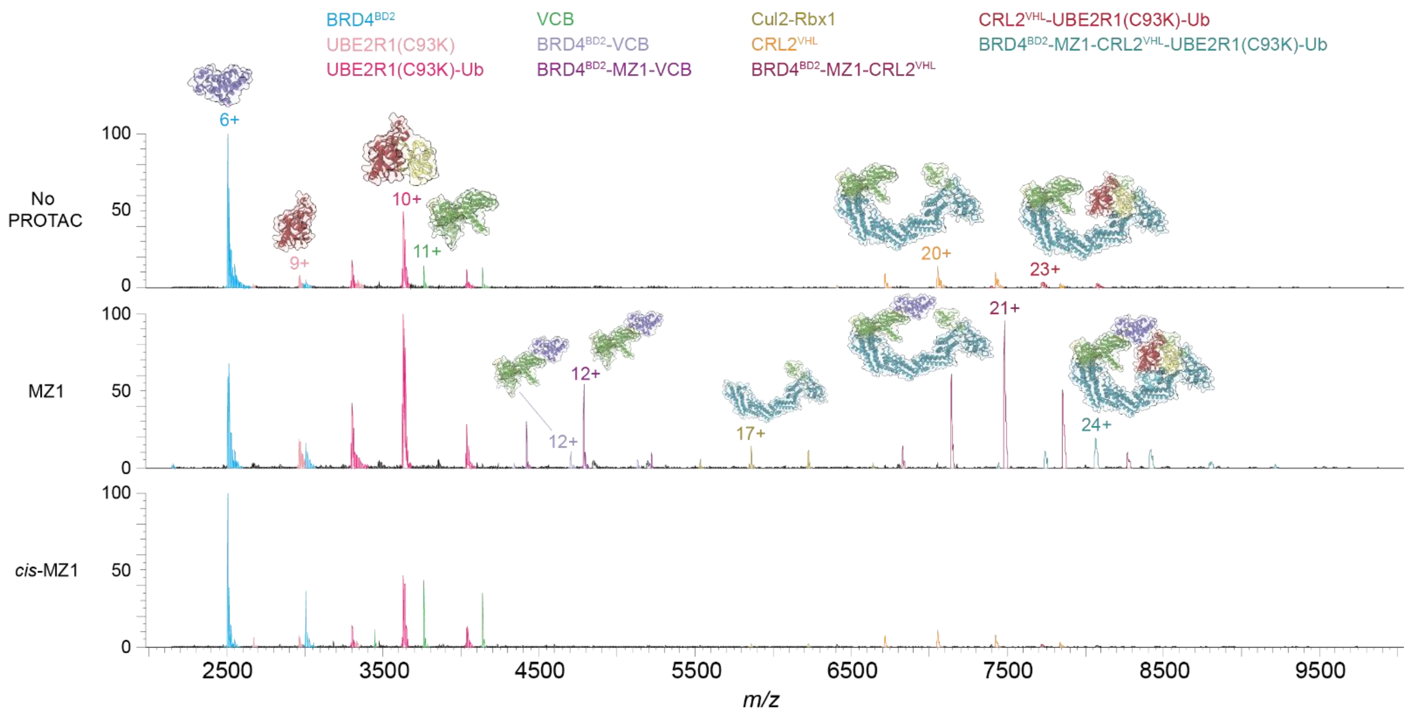

Supporting Figure S6. Raw data nMS spectra showing PROTAC mediated formation of the full BRD4^{BD2}-MZ1-CRL2^{VHL}-UBE2R1(C93K)-Ub complex. The peaks for each observed species that were present at >3% relative intensity following deconvolution are labelled via the indicated colour coding, and the charge state for the most intense peak for each observed species is also labelled. Corresponding deconvoluted nMS are shown in **Figure 5B-D** and observed *m/z* values and MWs provided in **Supporting Table S8**.

Supporting Table S8. Observed *m/z* values and measured MWs for the full POI-PROTAC-E3-E2-Ub degradation complex with $BRD4^{BD2}$ as the POI. Raw and deconvoluted mass spectra are shown in **Supporting Figure S6** and **Figure 5B-D**, respectively). Binary complexes – blue text, ternary complexes – red text, full complex in green text.

Sample Proteins	Compound	Protein Species	Expected MW (Da)	Observed *m/z*	Charge State (z)	Deconvoluted MW (Da)[a]
$BRD4^{BD2}$, $CRL2^{VHL}$, UBE2R1(C93K)-Ub	-	$BRD4^{BD2}$	15,036.3	2506.9248<br>3008.4526	6+<br>5+	15,036
		UBE2R1(C93K)	26,630.6	2669.9870<br>2959.9446<br>3337.5135	10+<br>9+<br>8+	26,631
		UBE2R1(C93K)-Ub	36,247.0	3301.6857<br>3631.8879<br>4035.2731	11+<br>10+<br>9+	36,308
		VHL-EloB-EloC	41,373.4	3762.2789<br>4138.4433	11+<br>10+	41,374
		$CRL2^{VHL}$	141,007.0	6409.1024<br>6715.4128<br>7051.9532<br>7424.0482<br>7838.6688	22+<br>21+<br>20+<br>19+<br>18+	141,020
		$CRL2^{VHL}$-UBE2R1(C93K)-Ub	177,254.0	7721.0020<br>8072.8616	23+<br>22+	177,589
	MZ1	$BRD4^{BD2}$	15,036.3	2149.0679<br>2506.8830<br>3008.4415	7+<br>6+<br>5+	15,037
		UBE2R1(C93K)	26,630.6	2959.9261	9+	26,629
		UBE2R1(C93K)-Ub	36,247.0	3301.6631<br>3631.7703<br>4035.2154	11+<br>10+<br>9+	36,308
		$BRD4^{BD2}$-VHL-EloC-EloB	56,409.7	4340.2769<br>4702.2645<br>5129.5862	13+<br>12+<br>11+	56,415
		$BRD4^{BD2}$-MZ1-VHL-EloC-EloB	57,412.3	4417.6583<br>4785.8801<br>5220.9530	13+<br>12+<br>11+	57,417
		Cul2-Rbx1	99,458.2	5533.2471<br>5858.9179<br>6225.1277<br>6716.1190	18+<br>17+<br>16+<br>15+	99,583

	BRD4^{BD2}-MZ1-CRL2^{VHL}	157,045.9	6829.0166	23+	157,070
			7140.0173	22+	
			7480.3888	21+	
			7855.3868	20+	
			8269.2690	19+	
	BRD4^{BD2}-MZ1-CRL2^{VHL}-UBE2R1(C93K)-Ub	193,292.9	7446.4773	26+	193,479
			7738.4118	25+	
			8064.4410	24+	
			8419.5551	23+	
			8795.9065	22+	
			9217.7988	21+	
cis-MZ1	BRD4^{BD2}	15,036.3	2506.9647	6+	15,036
			3008.1412	5+	
	UBE2R1(C93K)	26,630.6	2959.9504	9+	26,630
	UBE2R1(C93K)-Ub	36,247.0	3301.7445	11+	36,310
			3631.9606	10+	
			4035.5041	9+	
	VHL-EloB-EloC	41,373.4	3448.6484	12+	41,372
			3761.9328	11+	
			4138.4838	10+	
	Cul2-Rbx1	99,458.2	5532.6504	18+	99,584
			5858.6977	17+	
			6225.2509	16+	
	CRL2^{VHL}	141,007.0	6409.3917	22+	141,038
			6715.605	21+	
			7052.3944	20+	
			7424.4538	19+	
			7837.0402	18+	
	CRL2^{VHL}-UBE2R1(C93K)-Ub	177,254.0	7399.0569	24+	177,465
			7715.8799	23+	
			8067.6858	22+	
			8452.0352	21+	

[a]MW deconvoluted in UniDec,[2] to nearest whole Da and with relative intensity >3%.

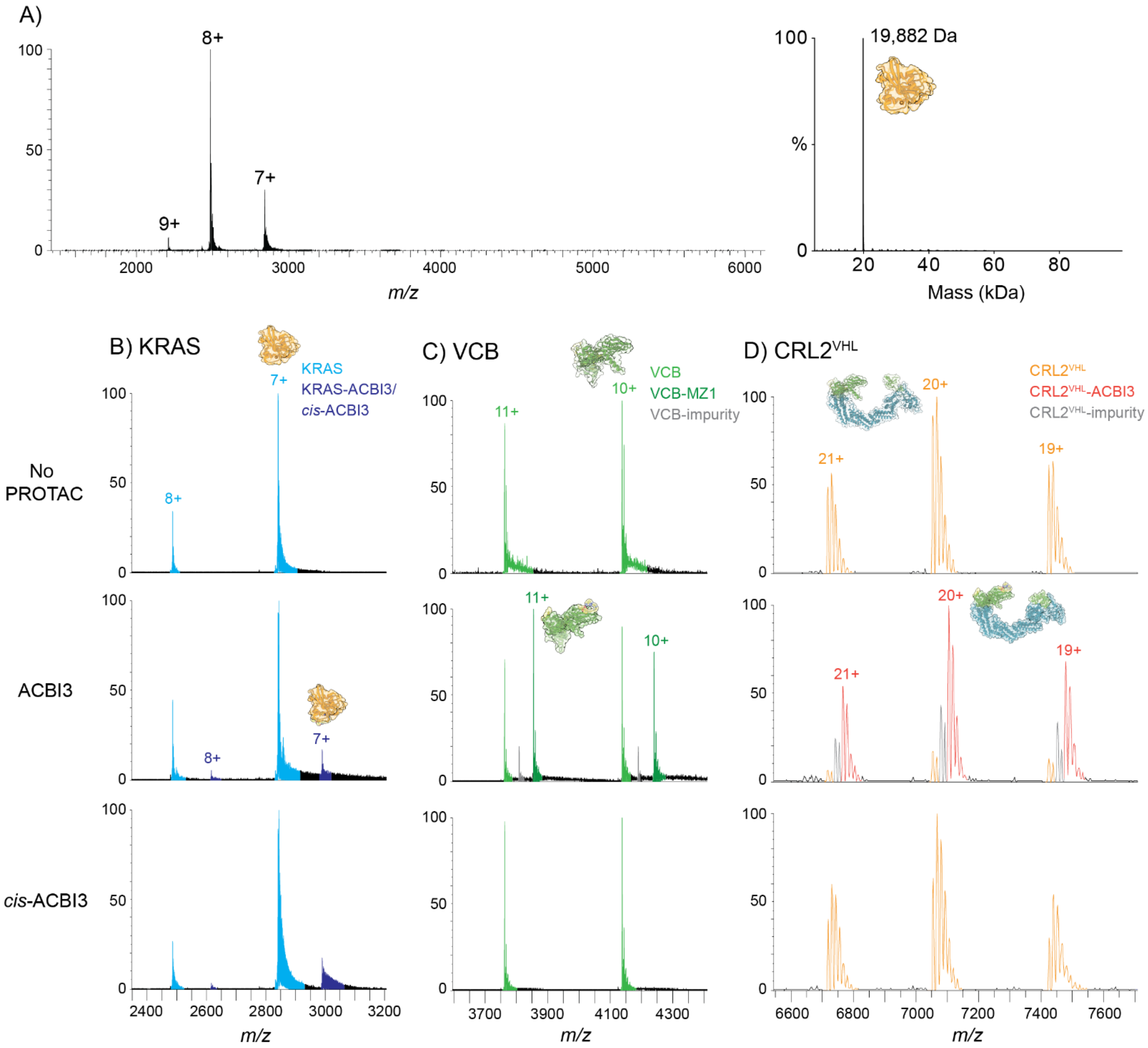

Supporting Figure S7. nMS assessment of KRAS and binary binding of ACBI3 and *cis*-ACBI3. A) Raw (LHS) and deconvoluted (RHS) nMS spectra (150 mM NH_4OAc) for 1 µM KRAS, expected MW is 19,850.4 Da, observed MW is 19,882 Da. B-D) Assessment of binary binding of PROTAC ACBI3 and negative control compound *cis*-ACBI3 (2 µM, 2 equiv) to 1 µM B) KRAS, C) VCB and D) CRL2^{VHL} (150 mM NH_4OAc, 1% DMSO). An impurity in the ACBI3 sample (MW ~515 Da) was also observed binding to VCB and CRL2^{VHL} but not to KRAS. The observed MW of 19,882 Da for KRAS is used for data analysis throughout the rest of the study. Observed *m/z* values and MWs are in **Supporting Table S9.**

Supporting Table S9. Observed *m/z* values and measured MWs for binary binding of ACBI3 (MW 1019.3 Da) and *cis*-ACBI3 (MW 1019.3 Da) to KRAS, VCB and CRL2^{VHL}. Raw spectra are shown in **Supporting Figure S7**. Binary complexes – blue text.

Sample		Protein Species	Expected MW (Da)	Observed *m/z*	Charge State (z)	Deconvoluted MW (Da)[a]	Bound Ligand MW (ΔMW) (Da)
**Protein**	**Ligand**						
KRAS	-	KRAS	19,882.0	2486.2000<br>2841.3773<br>3314.6055	8+<br>7+<br>6+	19,882	N/A
	ACBI3	KRAS	19,882.0	2486.4516<br>2841.3788<br>3318.2690	8+<br>7+<br>6+	19,883	N/A
		KRAS-ACBI3	20,901.3	2616.2527<br>2990.0114	8+<br>7+	20,924[b]	1,041[b]
	cis-ACBI3	KRAS	19,882.0	2486.1989<br>2841.236<br>3318.5996	8+<br>7+<br>6+	19,904[b]	N/A
		KRAS-*cis*-ACBI3	20,901.3	2616.5008<br>2990.0069<br>3488.1714	8+<br>7+<br>6+	20,923[b]	1,019
VCB	-	VCB	41,373.4	3448.7130<br>3762.0868<br>4138.0160<br>4596.8628	12+<br>11+<br>10+<br>9+	41,372	N/A
	ACBI3	VCB	41,373.4	3448.5737<br>3762.0069<br>4138.1159<br>4597.9829	12+<br>11+<br>10+<br>9+	41,372	N/A
		VCB-impurity[c]	N/A	3491.6228<br>3808.9210<br>4189.6719	12+<br>11+<br>10+	41,887	515[c]
		VCB-ACBI3	42,392.7	3533.5743<br>3854.7044<br>4240.0195<br>4711.4084	12+<br>11+<br>10+<br>9+	42,392	1,020
	cis-ABCI3	VCB	41,373.4	3448.5666<br>3762.0910<br>4138.1639<br>4597.7962	12+<br>11+<br>10+<br>9+	41,373	N/A
CRL2^{VHL}	-	CRL2^{VHL}	141,007	6412.0768<br>6717.8703	22+<br>21+	141,060	N/A

			7053.8870	20+		
			7425.1089	19+		
			7837.7589	18+		
ACBI3	CRL2^{VHL}	141,007	6411.4279	22+	141,064	N/A
			6716.9211	21+		
			7054.3442	20+		
			7425.2074	19+		
			7838.0195	18+		
	CRL2^{VHL}-impurity[c]	N/A	6435.7387	22+	141,577	513[c]
			6742.2366	21+		
			7079.7158	20+		
			7452.6289	19+		
			7866.6185	18+		
	CRL2^{VHL}-ACBI3	142,026.3	6458.0264	22+	142,078	1,014
			6766.2734	21+		
			7104.8006	20+		
			7478.9288	19+		
			7894.5842	18+		
cis-ACBI3	CRL2^{VHL}	141,007	6412.8701	22+	141,080	N/A
			6718.8696	21+		
			7054.8233	20+		
			7426.1097	19+		
			7838.3417	18+		

[a]MW deconvoluted in UniDec,[2] to nearest whole Da and with relative intensity >10%. [b]Additional mass corresponds to the binding of either a Na^{+} (23 Da) or Mg^{2+} (24 Da) adduct. [c]Binding of an ~515 Da impurity in ACBI3 detected. N/A: not applicable as no ligand binding observed.

Supporting Table S10. Expected MW of KRAS protein complexes (based on corrected MW).

**Protein**	**Corrected MW (Da)**[a]
KRAS	19,882[b]
VCB	41,373[c]
CRL2^{VHL}	141,007[b]
UBE2R1(C93K)	26,631[c]
UBE2R1(C93K)-Ub	36,247[b]
**Compounds/PROTACs**	**MW (Da)**
ACBI3	1019.3
cis-ACBI3	1019.3
**Protein Complexes**	**Expected MW (Da)**[d]
KRAS-VCB[e]	61,255
KRAS-ACBI3-VCB[f]	62,274
KRAS-CRL2^{VHL}[e]	160,889
KRAS-ACBI3-CRL2^{VHL}[f]	161,908
CRL2^{VHL}-UBE2R1(C93K)-Ub	177,254
KRAS-CRL2^{VHL}-UBE2R1(C93K)-Ub[e]	197,136
KRAS-ACBI3-CRL2^{VHL}-UBE2R1(C93K)-Ub[f]	198,155

[a]Corrected MW is based on the nMS analysis for protein components (**Supporting Figure S1** and **S7**) and are used for data analysis throughout the rest of the study. [b]MW as determined from nMS visibility studies (**Supporting Figure S1** and **S7**) due to differences with that expected from protein sequence. [c]MW as determined from protein sequence (corresponds with MW as measured by nMS, **Supporting Figure S1**). [d]Expected MWs of protein complexes of interest for this study, calculated using the corrected MWs for individual protein components (rounded to nearest Da). [e]Complexes without PROTAC and not necessarily expected to form, but MWs provided to compare with spectra to confirm presence or absence. [f]Complexes containing *cis*-ACBI3 are not expected to form, however, due to the MW of *cis*-ACBI3, any such complexes would have the same MW as the ACBI3-containing complexes.

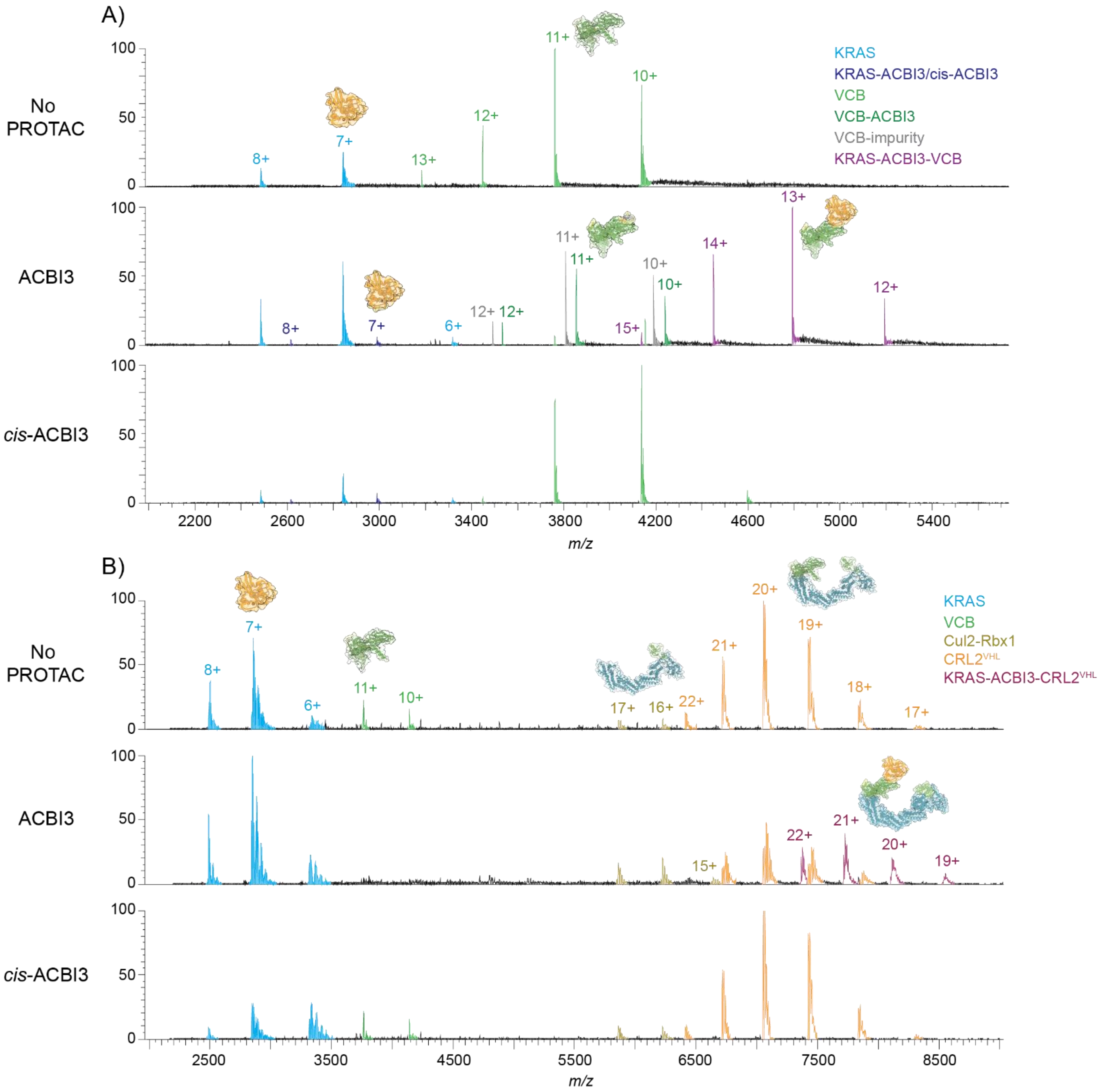

Supporting Figure S8. nMS analysis of ternary complex POI-PROTAC-E3 formation between KRAS (1 µM, 1 equiv), ACBI3 (1.9 µM, 1.9 equiv) and A) VCB (1 µM, 1 equiv), or B) CRL2^{VHL} (1 µM, 1 equiv) (150 mM NH_4OAc, 1% DMSO). Mass spectra are raw data with charge state and species identity annotated. Top panel is no PROTAC control (1% DMSO), middle panel is with addition of ACBI3, and lower panel is with addition of negative control *cis*-ACBI3 (1.9 equiv). Observed *m/z* values and MWs are in **Supporting Information**, **Table S11**.

Supporting Table S11. Observed *m/z* values and measured MWs for samples comprising KRAS and either VCB or CRL2^{VHL} with ACBI3 (MW 1019.3 Da) and *cis*-ACBI3 (MW 1019.3 Da). Raw mass spectra are shown in **Supporting Figure S8**. Binary complexes – blue text, ternary complexes – red text.

Sample POI	E3	Compound	Observed Species	Expected MW (Da)	Observed *m/z*	Charge State (*z*)	Deconvoluted MW (Da)[a]
KRAS	VCB	-	KRAS	19,882.0	2486.1421	8+	19,882
					2841.2028	7+	
			VCB	41,373.4	3183.4138	13+	41,372
					3448.6803	12+	
					3762.1409	11+	
					4138.2725	10+	
		ACBI3	KRAS	19,882.0	2486.1345	8+	19,882
					2841.1949	7+	
					3318.3043	6+	
			KRAS-ACBI3	20,901.3	2616.3069	8+	[b]
					2990.0156	7+	
			VCB	41,373.4	3761.9889	11+	[b]
					4138.2183	10+	
			VCB-impurity[c]	N/A	3222.9428	13+	41,887[c]
					3491.4963	12+	
					3808.9114	11+	
					4189.6723	10+	
			VCB-ACBI3	42,392.7	3261.9083	13+	42,391
					3533.5278	12+	
					3854.7394	11+	
					4240.1162	10+	
			KRAS-ACBI3-VCB	62,274.7	4154.0189	15+	62,297[d]
					4450.77	14+	
					4793.0842	13+	
					5192.4958	12+	
		cis-ACBI3	KRAS	19,882.0	2486.1345	8+	19,904[d]
					2841.1949	7+	
					3318.322	6+	
			KRAS-*cis*-ACBI3	20,901.3	2616.3069	8+	20,924[d]
					2990.0156	7+	
			VCB	41,373.4	3448.694	12+	41,372
					3762.1354	11+	
					4138.2702	10+	
					4597.9777	9+	
	CRL2^{VHL}	-	KRAS	19,882.0	2486.4315	8+	19,939[d]
					2841.4149	7+	
					3324.3709	6+	
			VCB	41,373.4	2762.2050	11+	41,372

			4138.3487	10+	
	Cul2-Rbx1	99,458.2	5862.8148<br>6227.2146	17+<br>16+	99,644
	CRL2^{VHL}	141,007	6412.7951<br>6718.7686<br>7054.806<br>7426.3355<br>7838.8268<br>8300.1577	22+<br>21+<br>20+<br>19+<br>18+<br>17+	141,078
ACBI3	KRAS	19,882.0	2486.4035<br>2841.48<br>3315.0734	8+<br>7+<br>6+	19,927[d]
	Cul2-Rbx1	99,458.2	5860.0158<br>6226.3478<br>6641.8889	17+<br>16+<br>15+	99,607
	CRL2^{VHL}	141,007.0	6717.7733<br>7054.015<br>7424.5654<br>7837.5147	21+<br>20+<br>19+<br>18+	141,048
	KRAS-ACBI3-CRL2^{VHL}	161,908.3	7364.7186<br>7716.3797<br>8102.8139<br>8543.4401	22+<br>21+<br>20+<br>19+	162,251
cis-ACBI3	KRAS	19,882.0	2486.3181<br>2841.5444<br>3318.497	8+<br>7+<br>6+	20,002[d]
	VCB	41,373.4	3762.205<br>4138.3487	11+<br>10+	41,373
	Cul2-Rbx1	99,458.2	5859.4455<br>6226.1817	17+<br>16+	99,595
	CRL2^{VHL}	141,007	6411.4829<br>6717.0906<br>7053.6539<br>7425.2275<br>7837.7281<br>8299.9313	22+<br>21+<br>20+<br>19+<br>18+<br>17+	141,059

[a]MW deconvoluted in UniDec,[2] to nearest whole Da and with relative intensity >5%. Increased mass compared to expected M corresponds to adducts such as Na^+ or Mg^{2+}. [b]Species not detected during deconvolution but evident in raw spectrum. [c]Binding of an ~515 Da impurity present in the ACBI3 sample observed. N/A: not applicable as exact impurity MW unknown.

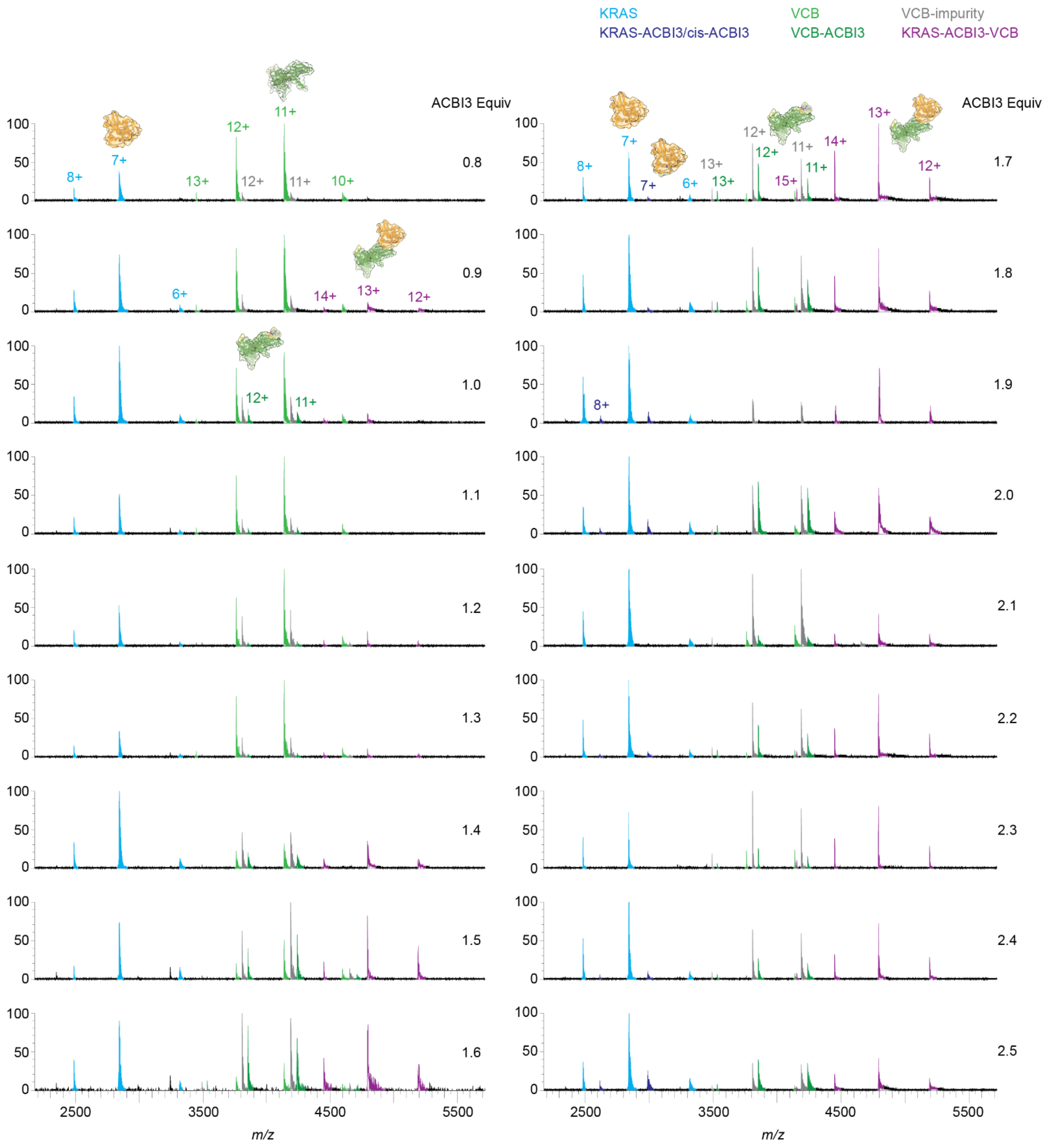

Supporting Figure S9. nMS titration of PROTAC ACBI3 (0.8-2.5 equiv, 0.1 increments) against POI KRAS and E3 VCB (present at equimolar concentrations, 1 μM each) to determine the optimal ACBI3 equiv for maximal ternary complex formation.

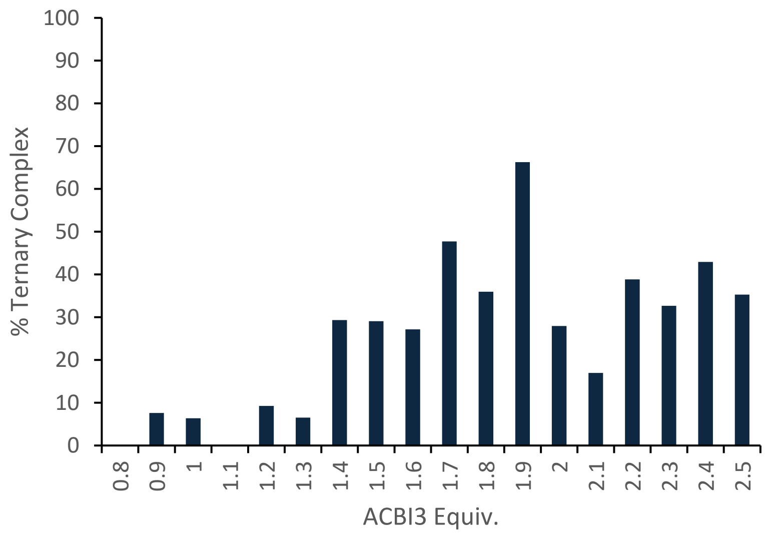

Supporting Figure S10. Quantification of ternary complex formation between KRAS and VCB (present at equimolar concentrations, 1 µM each) with ACBI3 titrated (0.8-2.5 equiv, 0.1 increments) as measured by nMS. The % ternary complex formed was calculated from the deconvoluted data of mass spectra in **Supporting Figure S9** as the ratio of VCB recruited into the ternary complex (i.e. sum of the intensity of the deconvoluted ternary complex peak divided by the sum of the intensities of the deconvoluted peaks for unbound VCB, impurity-bound VCB, ACBI3-bound VCB and ternary complex).

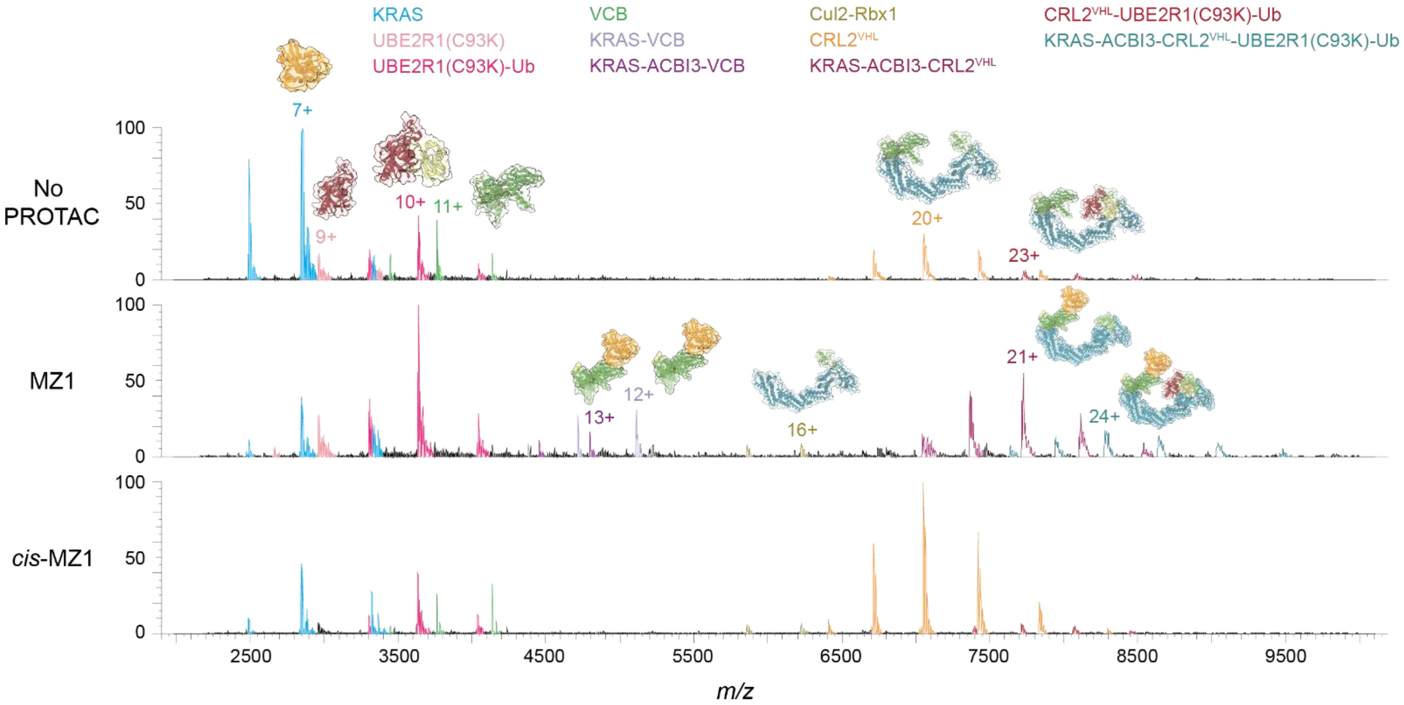

Supporting Figure S11. Raw data nMS spectra showing PROTAC mediated formation of the full KRAS-ACBI3-CRL2^{VHL}-UBE2R1(C93K)-Ub complex. The peaks for each observed species that were present at >3% relative intensity following deconvolution are labelled via the indicated colour coding, and the charge state for the most intense peak for each observed species is also labelled. Corresponding deconvoluted nMS are in manuscript **Figure 6D-F** and observed *m/z* values and MWs are provided in **Supporting Table S12**.

Supporting Table S12. Observed *m/z* values and measured MWs for the full POI-PROTAC-E3-E2-Ub degradation complex with KRAS as the POI. Raw and deconvoluted spectra are shown in **Supporting Figure S10** and **Figure 6D-F**, respectively). Binary complexes – blue text, ternary complexes – red text, full complex in green text.

Sample Proteins	Compound	Protein Species	Expected MW (Da)	Observed *m/z*	Charge State (*z*)	Deconvoluted MW (Da)[a]
KRAS, CRL2^{VHL}, UBE2R1(C93K)-Ub	-	KRAS	19,882.0	2486.2542	8+	19,904
				2841.3606	7+	
		UBE2R1(C93K)	26,630.6	2959.9899	9+	26,632
				3330.6674	8+	
		UBE2R1(C93K)-Ub	36,247.0	3301.6323	11+	36,307
				3631.6572	10+	
				4035.0980	9+	
		VHL-EloB-EloC	41,373.4	3448.6136	12+	41,373
				3762.2210	11+	
				4138.3816	10+	
		CRL2^{VHL}	141,007.0	6413.8685	22+	141,060
				6717.8919	21+	
				7053.7534	20+	
				7425.6475	19+	
				7838.8709	18+	
		CRL2^{VHL}-UBE2R1(C93K)-Ub	177,254.0	7728.0971	23+	177,734
				8080.9648	22+	
				8465.0562	21+	
	ACBI3	KRAS	19,882.0	2493.0915	8+	19,936
				2849.0872	7+	
		UBE2R1(C93K)	26,630.6	2663.9497	10+	26,630
				2959.9316	9+	
		UBE2R1(C93K)-Ub	36,247.0	3301.6092	11+	36,307
				3631.6854	10+	
				4035.2016	9+	
		KRAS-VCB	61,255.4	4380.4830	14+	61,317
				4717.5760	13+	
				5110.8157	12+	
		KRAS-ACBI3-VCB	62,247.7	4453.4398	14+	62,337
				4796.2024	13+	
		Cul2-Rbx1	99,458.2	5859.5129	17+	99,593
				6225.5701	16+	
		KRAS-ACBI3-CRL2^{VHL}	161,908.3	7044.6393	23+	162,023
				7365.2088	22+	
				7716.7247	21+	

			8103.2198	20+	
			8530.3381	19+	
	KRAS-ACBI3-CRL2^{VHL}-UBE2R1(C93K)-Ub	198,155.3	7638.5919	26+	198,644
			7944.2201	25+	
			8276.8724	24+	
			8642.0228	23+	
			9034.5203	22+	
			9459.2066	21+	
cis-ACBI3	KRAS	19,882.0	2486.3380	8+	19,882
			2841.3167	7+	
			3323.8015	6+	
	UBE2R1(C93K)-Ub	36,247.0	3301.6776	11+	36,307
			3631.7890	10+	
			4035.2441	9+	
	VHL-EloB-EloC	41,373.4	3448.5657	12+	41,374
			3762.2138	11+	
			4138.3817	10+	
	Cul2-Rbx1	99,458.2	5859.2432	17+	99,591
			6225.5027	16+	
	CRL2^{VHL}	141,007.0	6409.1202	22+	140,991
			6714.6777	21+	
			7050.4881	20+	
			7421.7902	19+	
			7834.6201	18+	
			8296.4481	17+	
	CRL2^{VHL}-UBE2R1(C93K)-Ub	177,254.0	7391.1594	24+	177,373
			7712.3778	23+	
			8063.4043	22+	
			8450.1208	21+	

[a]MW deconvoluted in UniDec,[2] to nearest whole Da and with relative intensity >3%. Increased mass compared to expected MW corresponds to adducts such as Na^{+} or Mg^{2+}.

Recombinant production of $CRL2^{VHL}$ protein.

50 µL of DH10Bac competent *E. coli* cells were thawed on ice. 1 µL (600 ng) plasmid DNA[1] was added and incubated on ice for 30 min. The cells were heat shocked at 42 °C for 90 seconds before incubating on ice for 5 min. 500 µL Luria Broth (LB) media was added under laminar flow and the cells were incubated at 37 °C for 5 h. 90 uL of the cell suspension was plated onto insect cell medium agar plates (containing 50 µg/mL kanamycin, 7 µg/mL gentamicin, 10 µg/mL tetracycline, 40 µg/mL IPTG and 100 µg/mL X-Gal) under laminar flow and incubated at 37 °C for 48 h.

5 mL of insect cell media (containing 50 µg/mL kanamycin, 7 µg/mL gentamicin, 10 µg/mL tetracycline) was inoculated with single white colonies. The freshly inoculated media was streaked onto new insect cell medium agar plates (containing 50 µg/mL kanamycin, 7 µg/mL gentamicin, 10 µg/mL tetracycline, 40 µg/mL IPTG and 100 µg/mL X-Gal) to discard false positives. The cultures were incubated at 37 °C with shaking at 180 rpm overnight. The plates were incubated at 37 °C for 48 h. The cells were pelleted and the bacmid was isolated through mini-prep. The bacmids were stored at 4 °C until transfection, before longer term storage at -20 °C. Successful recombination was verified by PCR.

Spodoptera frugiperda 9 (Sf9) insect cells were grown in suspension culture, using Gibco Sf-900™ II SFM media (ThermoFisher) supplemented with Gibco Antibiotic-Antimycotic (ThermoFisher). The cells were incubated at 27 °C with 130 rpm shaking, protected from light. The cells were split every 72 to 96 h to a density of 1.0×10^6 cells/mL. Cell count, viability and cell diameter were monitored using a Luna fl Cell Counter. The cell viability was maintained above 90% and the cell diameter of uninfected cells was usually 13-17 µm.

Sf9 insect cells were seeded at 1.0×10^6 cells/mL, one day prior to transfection. Once cells had reached 1.5×10^6 cells/mL, 5 mL of cells were seeded at 0.8×10^6 cells/mL into T25 flasks. The cells were allowed to attach for 15 min at room temperature. The media was aspirated and replaced with 3 mL of fresh Gibco Sf-900™ II SFM media. To a solution of 200 µL of Gibco Sf-900™ II SFM media and 8 µL of Cellfectin II (ThermoFisher), was added 3 µg of the bacmid DNA. The resulting mixture was mixed and incubated at room temperature for 30 min. 300 µL Gibco Sf-900™ II SFM media was added, and the transfection mixture was added to the cells dropwise. The culture was gently mixed, then incubated at 27 °C for 6 h. The media was aspirated and replaced with 5.5 mL of fresh Gibco Sf-900™ II SFM media supplemented with Gibco Antibiotic-Antimycotic. The culture was incubated for 6 days at 27 °C. The supernatant was collected and centrifuged at 1500 × g at room temperature for 5 min. The supernatant (P0 baculovirus stock) was collected, transferred to fresh flasks, and stored protected from light at 4 °C. For virus amplification, to a freshly seeded 300 mL culture of Sf9 cells at 1.0×10^6 cells/mL was added 1 mL of P0 baculovirus stock. The culture was incubated for 4 days at 27 °C with shaking at 130 rpm, protected from light. The culture was centrifuged at

800 × g at room temperature for 5 min. The supernatant (P1 baculovirus stock) was collected, transferred to fresh flasks, and stored protected from light at 4 °C.

Sf9 cells were seeded at a density of 1.0×10^6 cells/mL. After 24 h of incubation, the cells were infected with P1 baculovirus stock in a ratio of 1:50, 1:75 or 1:100. The culture was incubated for 72 hat 27 °C with shaking at 130 rpm, protected from light. The cells were harvested by centrifugation at 4000 rpm, 4 °C for 30 min. The cell pellets were resuspended in 50 mM HEPES, 250 mM NaCl, 2 mM TCEP and 0.2% (v/v) Triton-X, pH 8.0, flash frozen at stored at -80 °C until purification.

To the thawed and resuspended cell pellets was added cOmpleteTM EDTA-free protease inhibitor cocktail (Roche), 5 mM $MgCl_2$ and 10 µg/mL DNAse I. The mixture was incubated for 15 min at room temperature, and the cells were lysed at 15 kpsi on a Constant Cells cell disruptor. The protein-containing supernatant was clarified by centrifugation at 22,000 rpm for 20 min at 4 °C. Ampicillin-Sepharose resin was equilibrated in water, and the lysate was incubated with the resin for 50 min at room temperature. The resin was spun at 7000 rpm for 2 min, washed with 50 mM HEPES, 250 mM NaCl, 2 mM TCEP and 0.2% (v/v) Triton-X, pH 8.0, and was left in the same buffer. TEV protease was added along with an excess of purified recombinant VCB protein, and the protein was cleaved at room temperature for 2.5 h. The mixture was filtered, and the filtrate was purified on a 10/300 GL Superdex 200 Increase prepacked column (Cytiva) eluting in 20 mM HEPES pH 8.0, 100 mM NaCl, 5% (v/v) glycerol, 2 mM TCEP.

Recombinant production of KRAS protein.

KRAS (residues 1 to 169) wild type was expressed from a pET-s8a(+) expression vector and purified in *E. coli* BL21(DE3). An overnight culture was grown in LB supplemented with 50 µg/mL kanamycin at 37 °C, 180 rpm. To each 1L of fresh LB media (containing 50 µg/mL kanamycin), 10 mL overnight culture was added and further grown at 37 °C, 180 rpm. Cultures were grown until an OD600 of 0.8 was reached, after which cells were induced with 250 µM IPTG and the temperature was reduced to 18 °C. KRAS was expressed overnight and harvested the next morning by centrifugation at 4000 rpm, for 40 min, 4 °C using a Beckman Coulter Avanti J-HC equipped with a JS-4.2 rotor. The pellets were stored at -20 °C in approximately 200 mL of Buffer A (20 mM Tris, pH 8.0, 500 mM NaCl, 10 mM $MgCl_2$, 1 mM TCEP), supplemented with 2 tablets of cOmplete Protease Inhibitor Cocktail tablets (Roche) and 50 µL 140 µM benzonase. The pellets were thawed to room temperature prior to cell-disruption using a One Shot Cell Disrupter (Constant Systems) operating at 30 kpsi, 4 °C. The cell-debris was separated from the lysate by centrifugation at 25 000 rpm for 40 min at 4 °C, using a Beckman Coulter Avanti J-26S XP with a JA-25.50 rotor. The cleared lysate was syringe filtered through a 0.45 µm filter before running it over a Ni-affinity HisTrap HP column (Cytiva). The protein was eluted using a 10 to 300 mM imidazole gradient over 10 column volumes (CV) run at 5 mL/min in Buffer A. Fractions containing KRAS were combined and subjected to TEV protease cleavage (300 µL of

10 mg/mL TEV protease) while dialysing to Buffer A at 4 °C overnight. KRAS was further purified by running a reverse HisTrap using a 10 to 300 mM imidazole gradient over 10 CV run at 5 mL/min in Buffer A. Fractions containing KRAS were pooled and concentrated to a volume of 1.8 mL, after which it was further purified using gel filtration. A Cytiva HiLoad Superdex 75 pg 16/600 column was used at 0.35 mL/min, 4 °C using Buffer B (20 mM Tris, pH 7.5, 10 mM NaCl, 2 mM $MgCl_2$, 1 mM TCEP). Fractions containing pure KRAS were combined and concentrated to about 3 mg/mL. To the pure KRAS solution 0.5 mg of guanosine 5′-diphosphate (GDP) per mg of protein was added and incubated at 4 °C for 5.5 h. After incubation with GDP the buffer was exchanged to Buffer B without GDP and concentrated to 2 mM. 31 aliquots of 20 µL were flash frozen in liquid nitrogen and stored at -80 °C.

Supporting Table S13. UHMR MS parameters employed for the methods used to characterise proteins and/or complexes. Typically, 1-2 mins of acquired data were averaged per spectrum. nMS data were analysed directly using Themo Fisher Scientific FreeStyle 1.8 SP2 QF1 and deconvoluted in UniDec.[2]

Parameter	BRD4^{BD2} Binary Binding	KRAS Binary Binding	VCB Binary Binding	VCB Ternary Complexes	CRL2^{VHL} Binary Binding and CRL2^{VHL} Ternary Complexes	E3-E2 Interactions and BRD4^{BD2}-PROTAC-E3-E2-Ub Complexes	KRAS-PROTAC-E3-E2-Ub Complexes
Polarity	Positive	Positive	Positive	Positive	Positive	Positive	Postitive
nESI Source	NanoMate	NanoMate	NanoMate	NanoMate	Static	Static	Static
Spray voltage (V)	1.7	1.7	1.7	1.7	0.8-1.1	0.8-1.4	0.8-1.2
NM pressure (psi)	1.0	1.0	1.0	1.0	N/A	N/A	N/A
Capillary temperature (°C)	275	275	275	230	240	275	240
S-lens RF level	200	200	200	200	200	200	200
In-source trapping desolvation voltage (V)	-5	0	-5	-10	-60	-40	-60
Source DC offset (V)	5	21	5	21	21	21	21
Injection flatapole DC (V)	5	5	5	5	5	5	5
Inter flatapole lens (V)	4	4	4	4	4	4	4
Bent flatapole DC (V)	2	2	2	2	2	2	2
Transfer multipole DC (V)	0	0	0	0	0	0	0
C-trap entrance lens (V)	1.8	1.8	1.8	1.8	1.8	1.8	1.8

Ion transfer target *m/z*	High *m/z*	High *m/z*	High *m/z*	High *m/z*	High *m/z*	High *m/z*	High *m/z*
Trapping gas pressure	1.0	3.0	1.0	1.0	4.0	4.0	4.0
Extended trapping	1.0	1.0	1.0	1.0	10.0	10.0	10.0
Detector *m/z* optimisation	Low *m/z*	Low *m/z*	Low *m/z*	Low *m/z*	Low *m/z*	Low *m/z*	Low *m/z*
Scan range *m/z*	1,500-6,000	1,500-6,000	1,500-6,000	1,500-8,000	2,000-10,000	2,000-10,000	2,000-10,000
Resolution	200,000	100,000	25,000	12,500	6,250	6,250	6,250
Microscans	5	5	5	5	10	10	10
Manual inject time	5	100	100	100	200	200	200
Averaging	5	5	5	5	5	10	5
**UniDec Processing**							
m/z range	1,500-6,000	1,500-6,000	1,500-8,000	1,500-8,000	2,000-10,000	2,000-10,000	2,000-10,000
Charge state range	4-7	3-12	4-15	3-40	3-30 or 40	3-40	3-50
MW range (Da)	10,000-100,000	5,000-30,000	10,000-100,000	5,000-200,000	5,000-200,000	10,000-200,000 or 250,000	5,000-250,000
Sample mass every x Da	1	1	1	1	1	1	1
Peak detection range (Da)	500	500	500	500	500	500	500
Peak detection threshold (Da)	0.1	0.05	0.1	0.05	0.05 or 0.1	0.05 (lowered to 0.03 where required)	0.05 (lowered to 0.03 where required)

References

(1) Cardote, T. A. F.; Gadd, M. S.; Ciulli, A. Crystal Structure of the Cul2-Rbx1-EloBC-VHL Ubiquitin Ligase Complex. *Structure* **2017**, *25* (6), 901-911.e903. DOI: 10.1016/j.str.2017.04.009.
(2) Marty, M. T.; Baldwin, A. J.; Marklund, E. G.; Hochberg, G. K. A.; Benesch, J. L. P.; Robinson, C. V. Bayesian Deconvolution of Mass and Ion Mobility Spectra: From Binary Interactions to Polydisperse Ensembles. *Anal Chem* **2015**, *87* (8), 4370-4376. DOI: 10.1021/acs.analchem.5b00140.
